# Simultaneous Rupture of the Bladder and Pelvicaliceal System Because of Blunt Abdominal Trauma: A Rare Case

**DOI:** 10.1089/cren.2018.0051

**Published:** 2019-05-30

**Authors:** Sarosh Janardanan, Ravindra Kulkarni, Nimalan Arumainayagam

**Affiliations:** Department of Urology, Ashford and St Peter's NHS Trust Hospital, Chertsey, United Kingdom.

**Keywords:** bladder rupture, pelvicaliceal injury, blunt trauma, rendezvous stenting

## Abstract

We present a unique case of simultaneous rupture of the bladder and left renal pelvis after blunt trauma to the lower abdomen. To the best of our knowledge, this has not yet been reported in the literature. Another unusual aspect of this case was that the bladder rupture was bilateral, with both an extra- and intraperitoneal component. The management of this case was challenging. This involved an emergency laparotomy to repair the bladder tear, followed by a nephrostomy. This was followed by left ureteral stent insertion using a rendezvous technique. The case also highlights the role of expectant conservative management relating to the concurrent left renal pelvic rupture.

## Introduction and Background

The genitourinary system is involved in 10% of abdominal trauma.^1–3^ An underlying pathology predisposes to significant injury after trivial trauma and chronic outflow obstruction is often responsible for this presentation.^4^ The management of an intraperitoneal bladder injury involves surgical closure and catheter drainage, whereas most extraperitoneal bladder injuries heal well with bladder drainage alone.^1^ Retroperitoneal urinary extravasation or urinoma formation after a pelvicaliceal rupture often heals spontaneously and stent insertion is indicated if the extravasation persists.

## Presentation of Case

### Clinical history

A 67-year-old Caucasian man presented to the emergency department with a history of a fall, after he tripped while walking. He presented with abdominal pain and an inability to pass urine. He had no other significant past medical history apart from recently diagnosed hypertension.

### Physical examination

He was hemodynamically stable. His abdomen was distended and the lower abdomen was tender.

### Diagnosis

**FIG. 1.**
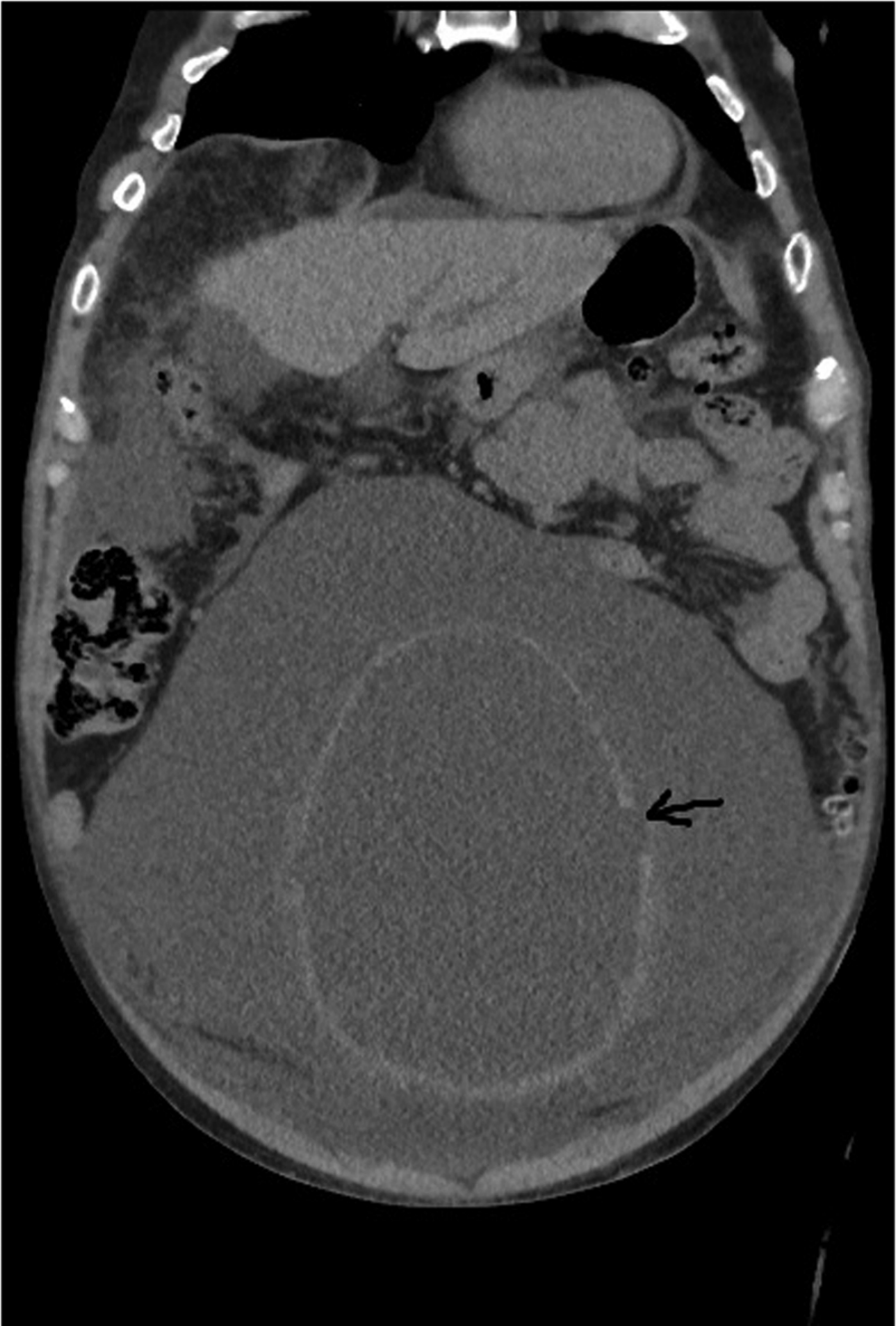
Coronal section of CT showing perforation (*arrow*) of the bladder wall with surrounding fluid collection.

**FIG. 2.**
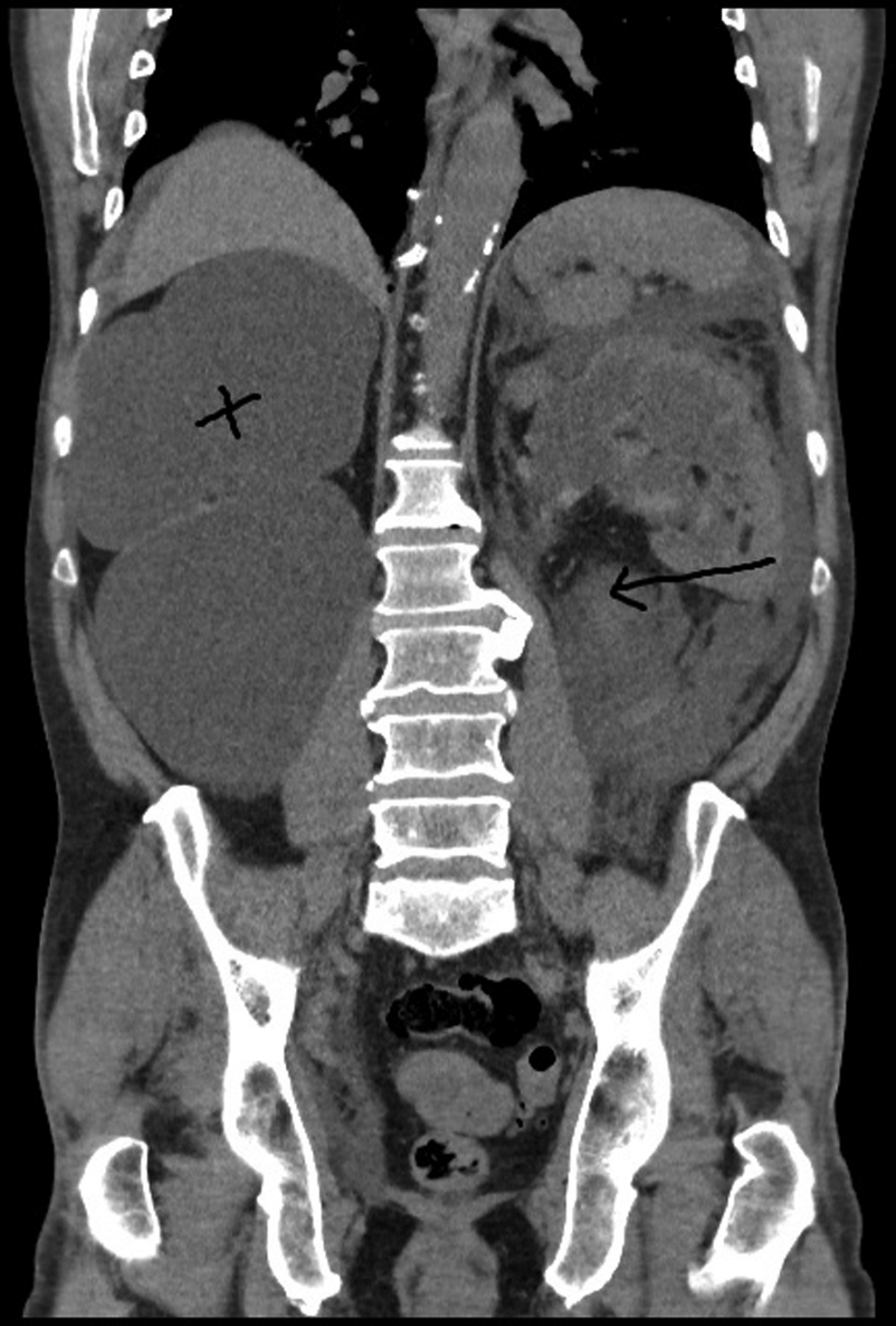
Coronal section of CT showing right nonfunctioning kidney (*cross*) with left retroperitoneal collection (*arrow*).

His blood results showed a white cell count of 15.4 × 10^9^/L, hemoglobin of 16.1 gm/dL, and creatinine of 464 mmol/L (previous blood tests were normal). A noncontrast CT scan revealed a large collection of fluid in the peritoneal cavity. The bladder was grossly distended with evidence of extraperitoneal fluid (Fig. 1). The right kidney was grossly hydronephrotic and had minimal cortex. The left functional kidney was enlarged consistent with compensatory hypertrophy, with a hydroureter (down to the level of the ureteral orifice) and hydronephrosis, with a multiloculated retroperitoneal collection suggesting secondary pelvicaliceal rupture (Fig. 2). The remaining abdominal organs appeared normal on CT imaging.

### Intervention

A midline laparotomy revealed a grossly distended bladder, with both extraperitoneal and intraperitoneal urine, and full thickness bilateral tears in the bladder. One tear was 8 cm long and the other 6 cm. Both were repaired in two layers using polyglactin sutures after draining the free fluid from the peritoneal cavity. The left ureteral orifice could not be identified on cystotomy and hence a retrograde study or stenting was not performed in this setting. Suprapubic and urethral catheters were left in place postoperatively.

**FIG. 3.**
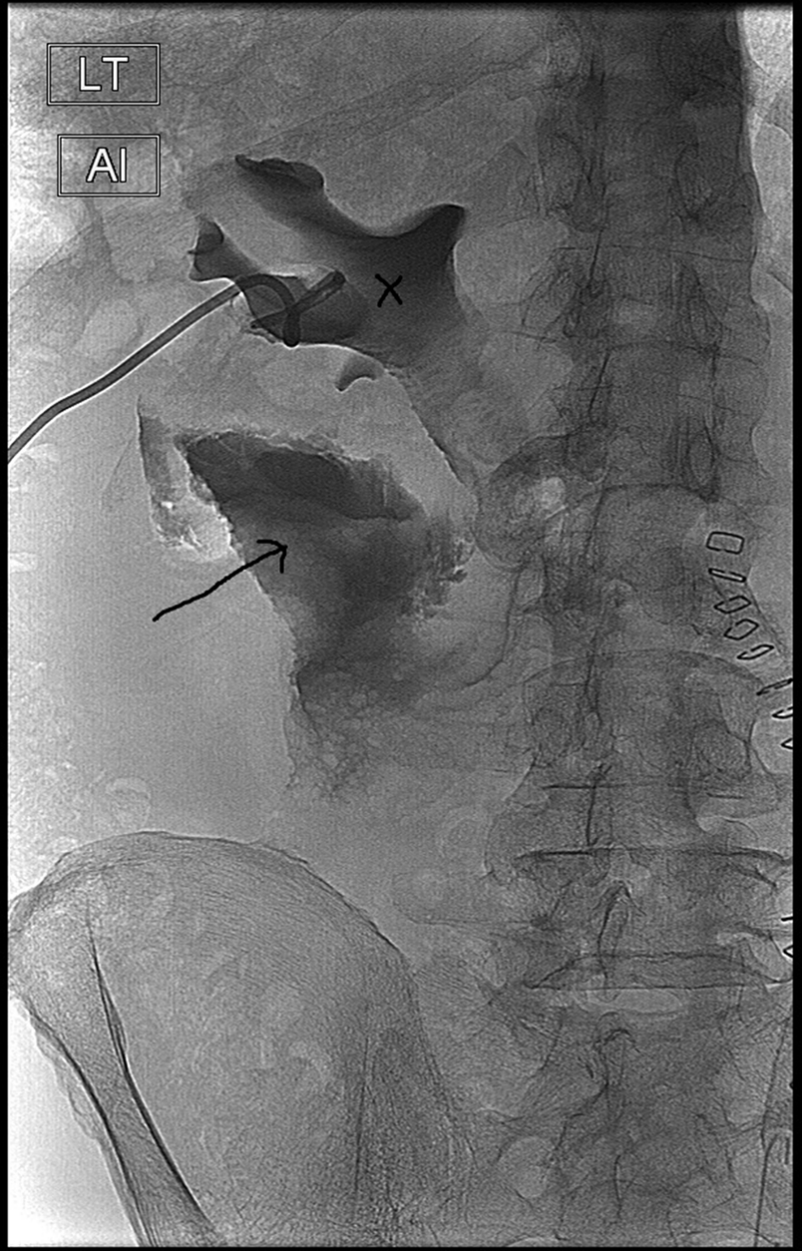
Left nephrostogram showing extravasation of contrast (*arrow*) with pelvicaliceal outline (*cross*) but no delineation of the ureter.

During the immediate postoperative period, a left nephrostomy (8F pigtail) was inserted under ultrasonography guidance. The patient recovered well with good urine output from the nephrostomy and normalization of his renal function. A nephrostogram revealed a grossly dilated pelvicaliceal collecting system with large retroperitoneal extravasation of contrast. The ureter was not delineated on this study (Fig. 3). A few days later an attempt was made to stent the left ureter trying both retrograde and antegrade approach. This was ineffective as the left ureteral orifice was not identified to allow retrograde access, and an antegrade wire insertion could not be negotiated into the proximal ureter because of the discontinuity.

### Follow-up

**FIG. 4.**
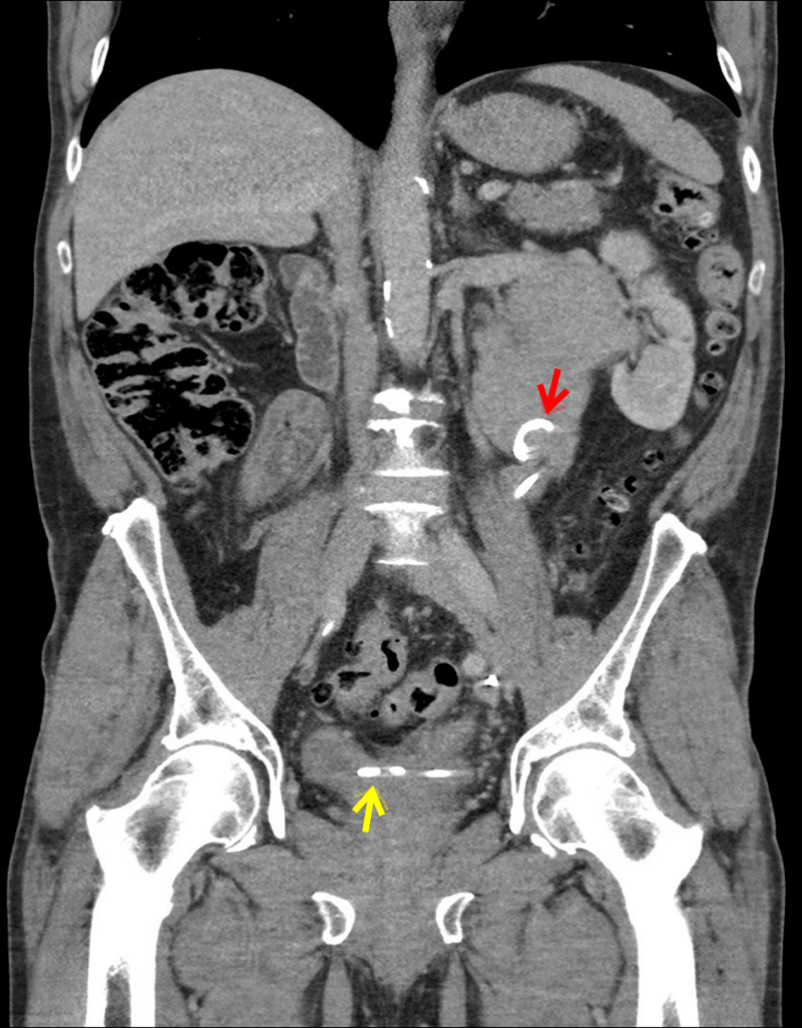
CT urogram showing stent (*red arrow* pointing to top end in dilated pelvis and *yellow arrow* to lower end in the bladder) in the baggy, distended system with no retroperitoneal collection.

An elective rendezvous procedure was planned after 4 weeks to allow the collection and inflammation to settle. A repeat CT confirmed reduction in the size of the retroperitoneal collection. During the rendezvous procedure, a Terumo hydrophilic guidewire was passed down to the bladder through an antegrade approach into the bladder and a stent (6F/26 cm) was secured in the left ureter (Fig. 4). The ureter was found to be grossly dilated and tortuous. The prostate was grossly enlarged.

### Outcomes

**FIG. 5.**
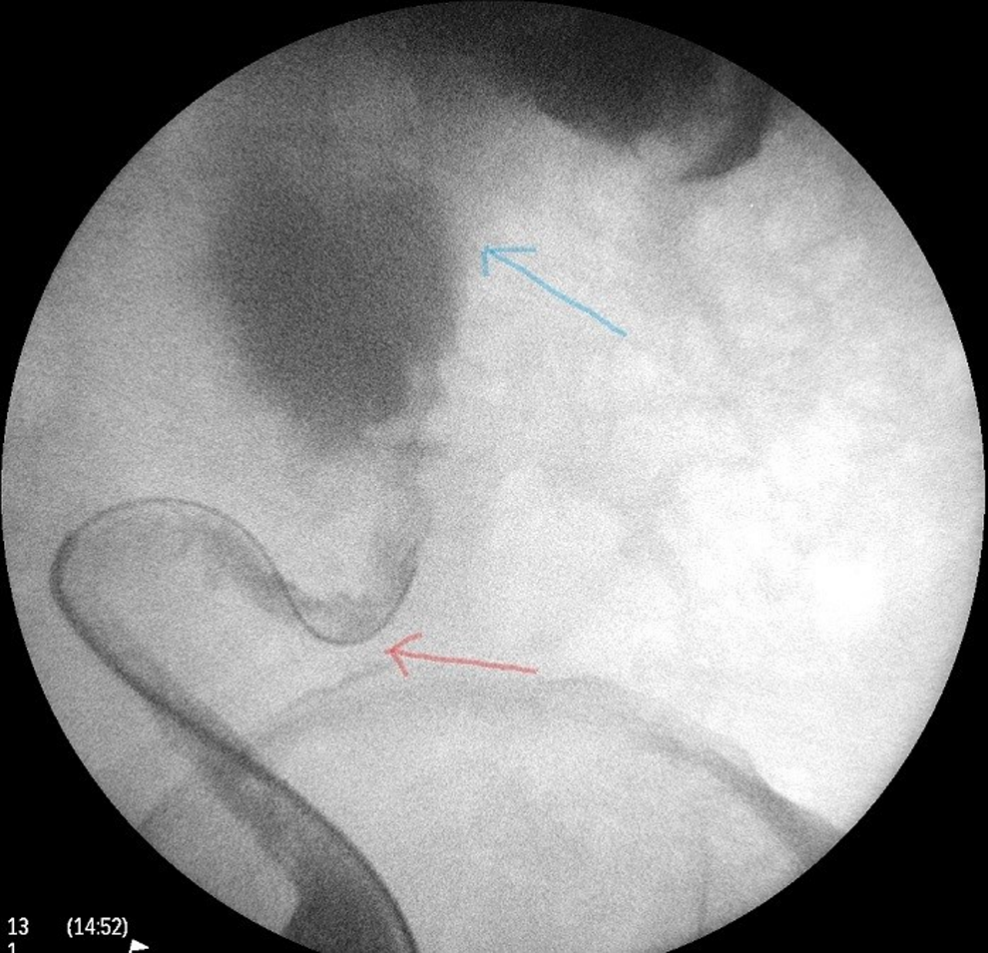
Retrograde study showing a tortuous dilated ureter (*red arrow*) and a baggy renal pelvis (*blue arrow*).

Reassessment after 4 weeks with a CT urogram showed satisfactory healing of both the bladder and upper tract injuries with no evidence of leak. This was confirmed on cystogram and retrograde study (Fig. 5). The Double-J stent was removed and the patient was discharged on a long-term suprapubic catheter because of the presence of an atonic floppy bladder. All his blood investigations have returned back to baseline.

## Conclusion

This case highlights how significant blunt force to a chronically distended bladder in the presence of “high-pressure” chronic retention (and thus a dilated ureter and collecting system) can result in both concurrent lower urinary tract and upper urinary tract rupture.

The nature of the injury involved the patient falling forward onto their chronically distended bladder, which took the impact of this fall. This resulted in bilateral bladder wall tears with simultaneous left pelvicaliceal rupture (“pop off phenomenon”) because of sideways transmission of force to the lateral walls of the bladder and also a transmission of this force up through the standing column of urine in the left hydroureter to the distended left renal pelvis (with resultant renal caliceal rupture).

Although injury to the lower urinary tract required laparotomy and surgical repair, the upper tract injury in this case seems to have settled with a period of conservative management after insertion of a nephrostomy tube and subsequent rendezvous ureteral stent insertion.

## Abbreviation Used

CT: computed tomography
